# The kinase inhibitor D11 induces caspase-mediated cell death in cancer cells resistant to chemotherapeutic treatment

**DOI:** 10.1186/s13046-015-0234-6

**Published:** 2015-10-20

**Authors:** Barbara Guerra, Mette Fischer, Susanne Schaefer, Olaf-Georg Issinger

**Affiliations:** Department of Biochemistry and Molecular Biology, University of Southern Denmark, Odense, Denmark; KinaseDetect Aps, Kruså, Denmark

**Keywords:** Kinase inhibitors, EGFR, NF-κB, NKCC1, ROCK, Apoptosis, Glioblastoma, Pancreatic adenocarcinoma

## Abstract

**Background:**

Multi-drug resistance and predisposition to metastasize are major clinical problems in cancer treatment. Malignant primary brain tumor and pancreatic cancer are two well-known examples of malignant tumors resistant to conventional therapies where aberrant EGFR-mediated and NF-κB signal transduction pathways are likely to play an important role. We have recently identified 1,3-Dichloro-6-[(E)-((4-methoxyphenyl)imino)methyl] diben-zo(b,d) furan-2,7-diol (D11) as a potent and selective inhibitor of CK2 a serine/threonine protein kinase that modulates the aforementioned signaling cascades.

**Methods:**

Human cancer cell lines (glioblastoma and pancreatic adenocarcinoma) resistant to conventional chemotherapeutic agents were incubated with increasing concentrations of D11 for variable amounts of time. Cell viability, cell death and effects on major signal transduction pathways deregulated in cancer cells were analyzed by ELISA, FACS and Western blot-based assays, respectively. Moreover, effects on cell migration and in cell protein-protein association were investigated by wound-healing and *in situ* proximity ligation assays, respectively.

**Results:**

We show here, that D11 treatment leads to i) significant caspase-mediated apoptotic cell death, ii) down-regulation of EGFR expression and iii) inhibition of NF-κB transcriptional activity. Furthermore, cell exposure to D11 results in impaired cell migration and correlates with reduced expression of the ion co-transporter and cell volume regulator Na^+^-K^+^-2Cl^−^ (NKCC1).

**Conclusions:**

Data reported here underline the therapeutic potential of D11 with respect to certain types of cancer that carry aberrant intracellular signaling cascades and/or exhibit sustained cell migration and suggest a new therapeutic strategy against chemotherapy resistance.

**Electronic supplementary material:**

The online version of this article (doi:10.1186/s13046-015-0234-6) contains supplementary material, which is available to authorized users.

## Background

Cancer is a disease characterized by genomic alterations that confer a selective growth advantage to cancer cells. In recent years, large-scale multi-dimensional analysis of clinical data has provided a detailed map of the major molecular alterations occurring during cancer development and led to two important observations: i) tumors originating from the same type of tissue or organ display significant differences with respect to genomic alterations and ii) tumors with different origin bear common genetic alterations that result in enhanced survival, proliferation and drug resistance [1, 2]. The epidermal growth factor receptor (EGFR) signaling is a major regulator of cell survival and motility (reviewed in [3]). Overexpression or mutation of EGFR is often responsible for tumor resistance to both chemotherapy and radiotherapy [4–9]. Resistance of tumors overexpressing EGFR is linked to aberrant phosphoinositide 3-kinase (PI3K)/AKT pathway, a key signal transduction system that links multiple receptor classes and oncogenes [10, 11]. Recent cancer genomic studies have revealed that multiple components of the PI3K pathway are frequently targeted by germline or somatic mutations [12–14]. In this respect, the tumor suppressor phosphatase and tensin homolog (PTEN) and AKT are found frequently mutated in cancer ([15] and reviewed in [16]). The nuclear factor-κB (NF-κB) signaling pathway is often constitutively activated in cancer due to mutations in the genes coding for NF-κB isoforms or in those coding for proteins (e.g. IκBα) that control NF-κB activity [4, 6, 11].

We have recently identified 1,3-Dichloro-6-[(E)-((4-methoxyphenyl)imino)methyl] diben-zo(b,d) furan-2,7-diol (referred to as D11) as a novel potent and selective inhibitor of protein kinase CK2 from a screening of 1600 compounds of the Diversity Set III from the DTP program of the NCI/NIH [17]. Protein kinase CK2 is a constitutively active serine/threonine protein kinase whose expression and activity has been found elevated in all so far investigated tumors and highly proliferating tissues (reviewed in [18, 19]). Interestingly, CK2 modulates the PI3K/AKT signaling cascade by targeting AKT and PTEN for phosphorylation. Moreover, it regulates the NF-κB pathway by promoting IκB degradation and NF-κB phosphorylation that results in enhanced DNA binding and transcriptional activity of the latter [20–23].

In this study, we have analyzed the potency and biochemical mechanisms of cell death induction of D11 on glioblastoma and pancreatic adenocarcinoma cells that are characterized by aberrant EGFR/PI3K and NF-κB signaling pathways and resistance towards radio- and chemotherapy [24–27]. Results reported here show that D11 exerts potent anti-tumor effects warranting further *in vivo* studies for validating its efficacy against multi-drug resistant cancer cells.

## Materials and methods

### Cell culture and treatments

The human glioblastoma cell lines M059K and U-87 MG and the human pancreatic adenocarcinoma cell line MIA PaCa-2 were purchased from the American Type Culture Collection (ATCC, Rockville, MD, USA) and cultivated at 37 °C under a 5 % CO_2_ atmosphere in Dulbecco’s modified Eagle’s medium (DMEM, Invitrogen, Taastrup, Denmark) supplemented with 10 % fetal bovine serum (FBS, Biochrom AG, Berlin, Germany). MIA PaCa-2 cells were additionally cultivated in the presence of 2.5 % horse serum (Biochrom AG). Cells were treated with D11 (DTP, NIH/NCI, Rockville, MD, USA), IGF-1 (Calbiochem, Nottingham, UK) and TNFα (R&D Systems, Abingdon, UK) as indicated in the figure legends. Cell transfection was carried out with Lipofectamine 3000 reagent (Life Technologies, Naerum, Denmark) according to the manufacturer’s guidelines and a plasmid carrying the coding region for farnesylated AKT devoid of the PH domain prepared according to [28]. The correct sequence and orientation were verified by DNA sequencing. Neocarzinostatin (NCS) was kindly provided by Dr. Hiroshi Maeda, Kumamoto University, Japan.

### Determination of cell viability

D11-mediated cytotoxicity was determined by the WST-1 viability assay (Roche, Hvidovre, Denmark). Viability was quantified in a microtiter plate reader (VersaMax ELISA, Molecular Devices, Sunnyvale, CA, USA) after adding the WST-1 reagent to the cells according to the manufacturer’s guidelines.

### Flow cytometry analysis

Cell cycle analysis and determination of cell death was determined as previously described [29]. The analysis was carried out on a FACS-Calibur flow cytometer (BD Biosciences, San Jose, CA, USA). Acquired data were processed by Cell Quest Pro Analysis software (BD Biosciences). For each measurement, 10,000 events were analyzed.

### Preparation of whole cell lysate, Western blot analysis and antibodies

Cells were harvested and further processed for Western blot analysis as described in [26, 30]. The following primary antibodies were employed in the study: mouse monoclonal anti-AKT, mouse monoclonal anti-poly(ADPribose)polymerase (PARP), mouse monoclonal anti-RAFT1/FRAP/mTOR (all from BD Biosciences); mouse monoclonal anti-caspase 8, mouse monoclonal anti-caspase 9, rabbit monoclonal anti-caspase 3, rabbit polyclonal anti-PTEN, rabbit polyclonal anti-phospho-PTEN (S380/T382,383), rabbit monoclonal anti-phospho-AKT (S473), rabbit polyclonal anti-phospho-AKT (T308), rabbit polyclonal anti-phospho-mTOR (S2481), rabbit monoclonal anti-Raptor, rabbit polyclonal anti-phospho-Raptor (S792), rabbit monoclonal anti-Tuberin/TSC2, rabbit polyclonal anti-phospho-Tuberin/TSC2 (S1387), rabbit polyclonal anti-phospho-Tuberin/TSC2 (T1462), mouse monoclonal anti-phospho-p70S6K (T389), rabbit polyclonal anti-phospho-AMPKα (T172), rabbit polyclonal anti-AMPKα, rabbit monoclonal anti-NF-κB/p65/RelA, rabbit monoclonal anti-NF-κB/p65/RelA (S536), rabbit monoclonal anti-phospho-IKKα/β (S176,180), rabbit polyclonal anti-IKKα, rabbit polyclonal anti-IKKβ, rabbit monoclonal anti-phospho-IκBα (S32), mouse monoclonal anti-IκBα, rabbit monoclonal anti-NKCC1 (all from Cell Signaling Technology); mouse monoclonal anti-β-actin (Sigma-Aldrich); rabbit polyclonal anti-EGFR, rabbit polyclonal anti-p70S6K, rabbit polyclonal anti-HSP90, mouse monoclonal anti-CDC37 (all from Santa Cruz Biotechnology, Heidelberg, Germany); rabbit polyclonal anti-phospho-NKCC1 (T212,217), rabbit polyclonal anti-AKT1 (both from Millipore, Billerica, MA, USA) and rabbit polyclonal anti-phospho-NF-κB p65 (S529, Abcam, Cambridge, MA, USA).

### Immunostaining and *in situ* proximity ligation assay

Cells grown on coverslips were fixed with 4 % paraformaldehyde for 15 min, permeabilized with 0.1 % Na-citrate, 0.1 % Triton X-100, pH 7 for 5 min and, where indicated, counterstained with 4′,6′-diamidino-2-penylindole (DAPI, Sigma-Aldrich, Brøndby, Denmark). Actin filaments where visualized by Alexa Fluor 488 phalloidin staining (Invitrogen) for 20 min at room temperature according to the manufacturer’s recommendations. Localization of NF-κB was performed by incubating the cells with rabbit monoclonal anti-NF-κB/p65/RelA antibody (Cell Signaling Technology, Herlev, Denmark) at 4 °C overnight followed by labeling with biotinylated swine anti-rabbit immunoglobulin (Dako, Glostrup, Denmark) for 1 h at room temperature and Alexa Fluor 488-conjugated streptavidin (Life Technologies) for 30 min at room temperature. Cells were analyzed for HSP90-CDC37 interaction by *in situ* proximity ligation assay (PLA, Olink Biosciences, Uppsala, Sweden) according to the manufacturer’s recommendations. The number of positive signals was determined using Blobfinder software (http://www.cb.uu.se/~amin/BlobFinder). Cells were analyzed on a Leica DMRBE microscope equipped with a DFC 420C camera and Leica Application Suite V 3.3.0 software (Leica Microsystem, Wetzler, Germany).

### Transcription factor assay

The transcriptional activity of NF-κB was measured applying the TransAM assay system kit (Active Motif, Carlsbad, CA, USA) following the manufacturer’s recommendations. Quantification was performed with a VersaMax ELISA microplate reader (Molecular Devices).

### ROCK kinase assay

ROCK activity was measured using the ROCK1/2 activity assay kit (Cell Biolabs, Inc., San Diego, CA, USA). After treatment, cells were harvested, washed in ice-cold PBS and lysed. Kinase assay was performed according to the manufacturer’s recommendations. Measurements were obtained with a VersaMax ELISA microplate reader (Molecular Devices) using 450 nm as the primary wavelength.

### Wound-healing assay

Cells were cultured in 60 mm Petri dishes until confluent. Subsequently, straight scratches were made with a p200 pipet tip across the Petri dishes simulating a wound [31]. Pictures were taken at different intervals as indicated in the figure legends using phase contrast and the 5x objective on a DMIRB microscope (Leica Microsystem). Unhealed areas were quantified using the ImageJ64 software.

### Statistical and densitometric analysis

The statistical significance of differences between means of two groups was determined by the two-tailed *t*-test (Student’s *t*-test). The levels of significance are indicated in the figure legends.

## Results

### Cytotoxic effects of D11 in cancer cell lines

**Fig. 1:**
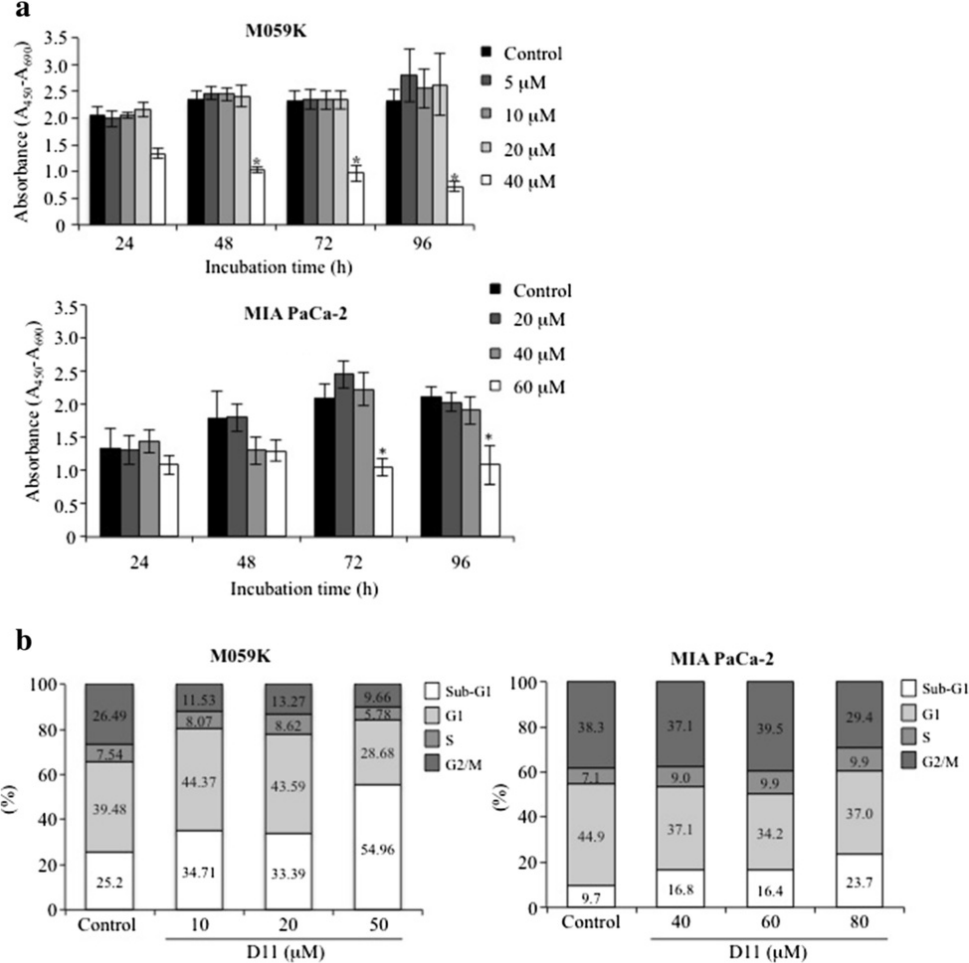
Anti-proliferative effects of D11 in human cancer cell lines. **a** M059K and MIA PaCa-2 cell lines were treated with increasing concentrations of D11 for a variable amount of time, respectively. Control experiment refers to cells treated with vehicle (0.1 % DMSO). The proportion of viable cells was determined by WST-1 assay and expressed in arbitrary units as a difference in absorbance measured at 450 nm and 690 nm (reference) wavelengths, respectively (mean +/− standard deviation, *N* = 6). Asterisks denote statistical significant differences between control and D11-treated cells for the corresponding time points, *, *P* <0.0001. **b** Flow cytometry analysis of cells treated with 0.1 % DMSO (Control) and increasing concentrations of D11 for 24 h (M059K) and 48 h (MIA PaCa-2), respectively. The amount of cells in the various phases of the cell cycle is indicated in percentage. Experiments were repeated three times obtaining similar results. Data from one representative experiment are shown

Human glioblastoma cells (M059K) and human pancreatic adenocarcinoma cells (MIA PaCa-2) were tested for *in vitro* sensitivity to D11 (Fig. 1a). The inhibitory concentration (IC_50_) was achieved with 40 μM D11 after 48 h of incubation in M059K cells while for MIA PaCa-2 cells, similar effects were achieved with 60 μM D11 after 72 h of incubation with respect to control experiments. Additionally, analysis of U-87 MG glioblastoma cells showed that treatment with 25 μM D11 for 24 h resulted in 50 % decrease in viable cells (Additional file 1: Figure S1).

**Fig. 2:**
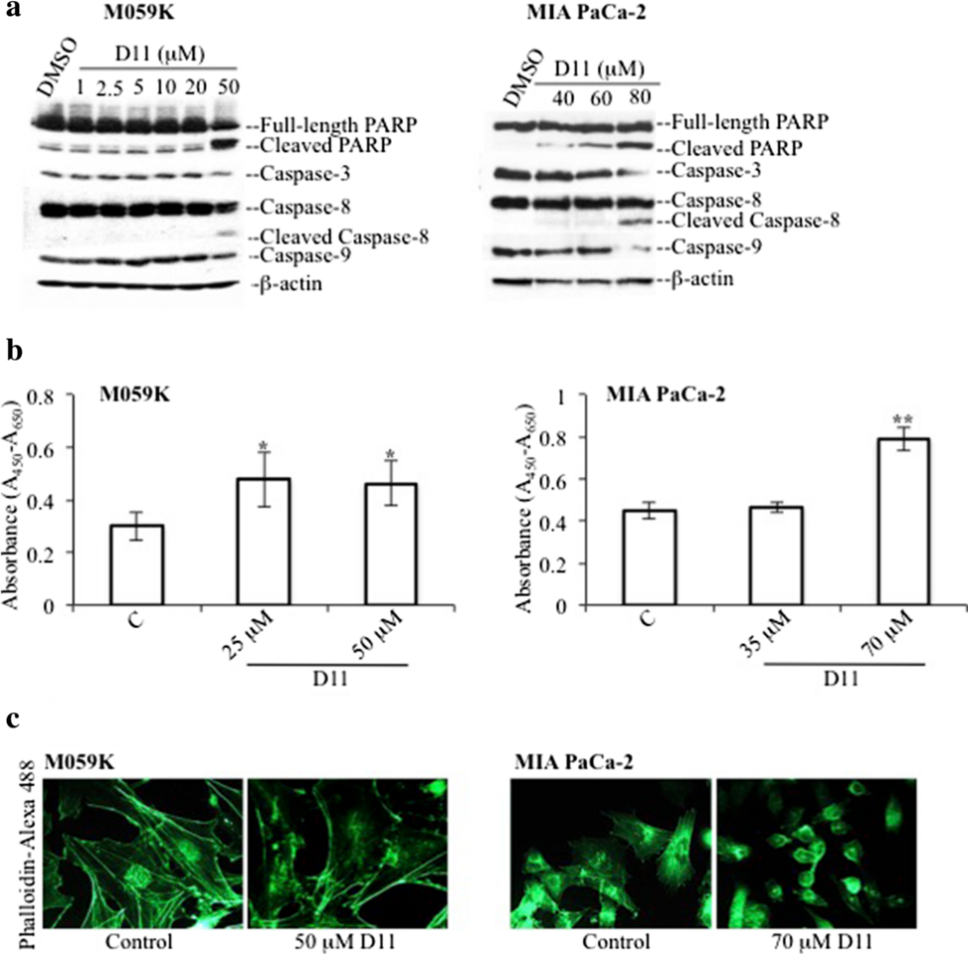
Exposure of cells to D11 leads to induction of apoptotic cell death. **a** Cells were treated with DMSO and increasing concentrations of D11 for 24 h (M059K) and 48 h (MIA PaCa-2), respectively. Whole cell lysate was subjected to Western blot analysis for apoptosis-associated proteins. **b** Whole cell lysate from cells treated for 24 h with the indicated concentrations were subjected to ROCK kinase assay in the presence of myosin phosphatase target subunit 1 (MYPT1). Data from one representative experiment (mean +/− standard deviation, *N* = 6, *, *P* <0.05; **, *P* <0.001) are shown. **c** Immunofluorescence analysis of control cells (0.1 % DMSO) or cells treated with the indicated concentrations of D11 for 24 h. Actin filaments were visualized by staining cells with phalloidin-Alexa 488. Representative images were acquired using fluorescence microscopy at 400x magnification

Next, cell cycle analysis was investigated by flow cytometry (Fig. 1b and Additional file 2: Figure S2). Cells were treated with various amounts of drug for 24 h (M059K) and 48 h (MIA PaCa-2), respectively. With respect to control experiments, significant differences in the percentage of cells with reduced DNA levels (i.e. sub-G1) were observed with 50 μM D11 (M059K) and 80 μM D11 (MIA PaCa-2) treatment, respectively. Western blot analysis (Fig. 2a) indicated that 50 μM D11 induces cleavage of full-length PARP in M059K cells after 24 h incubation time while significant PARP cleavage was observed in MIA PaCa-2 cells when treated with 60 and 80 μM D11 for 48 h, respectively. Analysis of cleavage-mediated caspase activation revealed that both the intrinsic and extrinsic apoptotic pathways [32] are involved in D11-induced cell death. In order to determine whether treatment with D11 can sensitize cancer cells towards conventional antitumor drugs, we incubated M059K with the radiomimetic drug neocarzinostatin (NCS) alone or in combination with D11. Combined treatment affected the viability (Additional file 3: Figure S3a) and resulted in enhanced apoptotic cell death (Additional file 3: Figure S3b) suggesting that D11 partially sensitizes glioblastoma cells to NCS.

Rho-associated kinase ROCK1 is a serine/threonine kinase that has been identified as a caspase-3 target involved in the blebbing process observed in apoptosis [33, 34]. Hence, we determined whether D11 treatment results in enhanced ROCK kinase activity. Significant increase in the phosphorylation of myosin phosphatase target subunit 1 (MYPT1) was observed as compared to control experiments, demonstrating that cell exposure to D11 leads to activation of ROCK kinase in both cell types (Fig. 2b). It has been shown that cells committed to detachment as observed in the activation of cell death, often exhibit disruption of central stress fibers and a contractile ring containing filamentous actin (F-actin) at the cell periphery [35]. Studies performed by employing mouse embryonic fibroblasts showed that cytoskeleton reorganization is dependent on the expression of active ROCK1 [36, 37]. Hence, we analyzed D11-mediated effects on actin filaments dynamics by staining cells with phalloidin [38]. D11 treatment resulted in actin cytoskeleton remodeling in both cell lines characterized by reduction of central stress fibers and formation of a ring of filaments at the cell periphery (Fig. 2c). Overall, results show that D11 exposure induces apoptotic cell death at varying treatment conditions depending on the type of cells, and that glioblastoma cells are more sensitive to the cytotoxic effects of D11 than pancreatic cancer cells.

### D11 treatment suppresses pro-survival signaling pathways

#### PI3K/AKT pathway

**Fig. 3:**
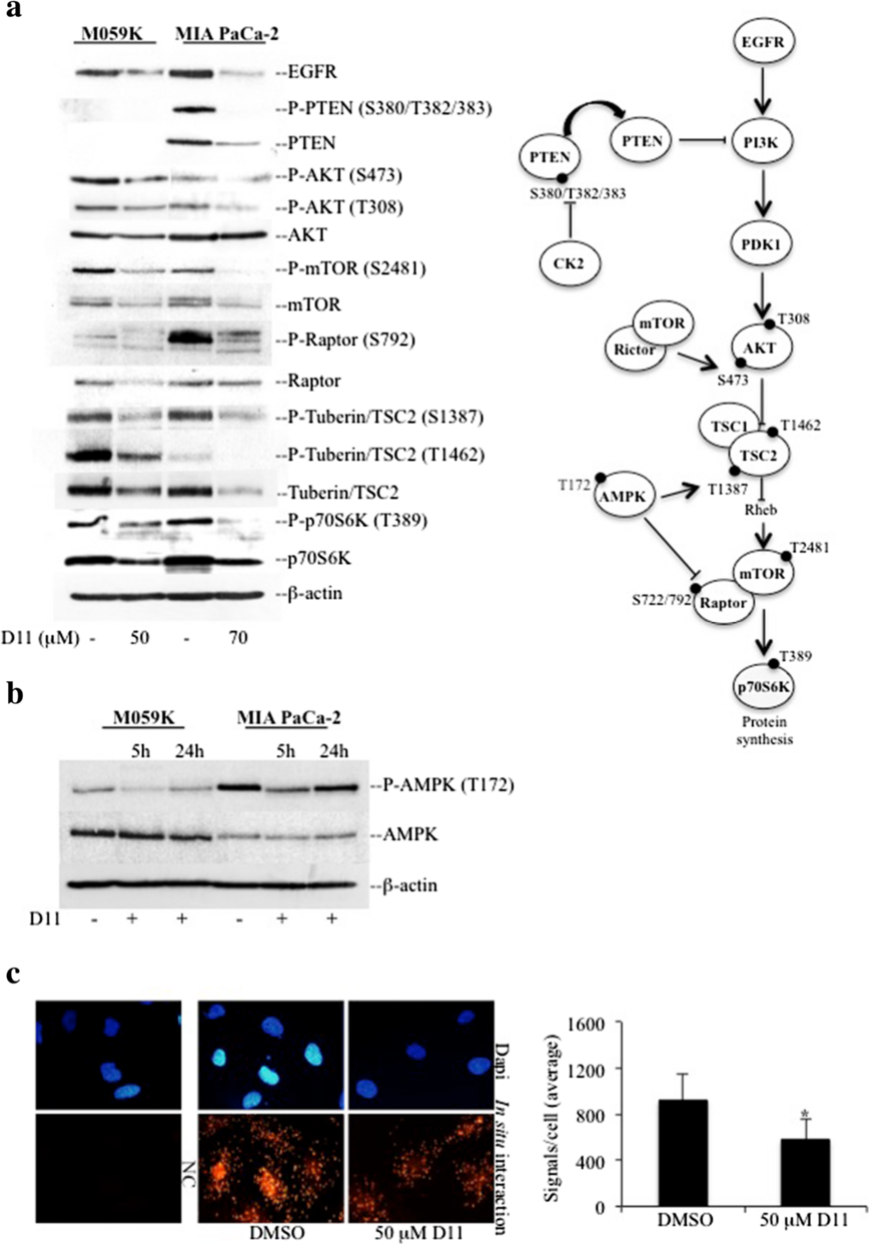
Analysis of the PI3K pathway in cells treated with D11. **a** Whole cell lysates from the indicated cell lines were subjected to Western blot analysis. Expression and/or phosphorylation of the indicated proteins were analyzed after 5 h incubation with D11. Control experiments (−) refer to cells incubated with vehicle (0.1 % DMSO). **b** Cells were treated with vehicle (−) or 50 μM (M059K) and 70 μM (MIA PaCa-2) D11, respectively, for the indicated times. Whole cell lysates were subjected to Western blot analysis of phosphorylated AMPK and AMPK protein. β-actin detection was used as a control for equal loading. **c** Association between HSP90 and CDC37 was revealed in M059K by *in situ* proximity ligation assay (PLA). NC refers to untreated cells subjected to PLA where one of the primary antibodies was omitted. Determination of the number of signals per cell as distinct red fluorescence spots indicative of HSP90-CDC37 proximity, was performed by computer-assisted image analysis as described in the Materials and methods. Nuclei were visualized by DAPI staining (blue fluorescence emission). Experiments were repeated at least three times obtaining similar results. The figure includes a schematic representation of the EGFR/PI3K signaling network in mammalian cells (*, *P* <0.05)

Components of the PI3K/AKT pathway were analyzed following short exposure to D11. Results presented in Fig. 3a show that D11 treatment led to a significant decrease of EGFR, mTOR, Raptor and Tuberin/TSC2 protein expression. This correlated with low phosphorylation levels of the corresponding proteins with respect to control experiments. Additionally, D11 treatment resulted in inhibition of AKT and p70S6K kinase activity although a less pronounced decrease in their expression levels was observed. PTEN status was investigated only in MIA PaCa-2 cells. According to Sonkin et al., M059K cells have a G-M PTEN status assigned to cell lines with homozygous nonsense, frame shift, known loss of function missense mutation or dominant negative mutation of a specific tumor suppressor gene [39]. The analysis revealed that D11 treatment leads to decreased PTEN expression and phosphorylation levels.

AMP-activated protein kinase (AMPK) is a nutrient sensor responsible for cellular energy homeostasis (reviewed in [40]). AMPK exerts dual regulatory effects on the PI3K pathway by targeting Tuberin/TSC2, an inhibitor of mTOR [41], and the TORC1 scaffold protein Raptor [42] for phosphorylation. Short exposure to D11 led to significant loss of AMPK kinase activity, which was partially recovered after 24 h of incubation time (Fig. 3b).

The chaperon protein HSP90 (heat shock protein 90) promotes folding, trafficking and stability of more than 200 client proteins including ErbB2, EGFR, Raf, MEK, FAK, PTEN, AKT, Raptor and mTOR [43–46]. Chaperones’ function is dependent on the presence of co-chaperone proteins and co-activators [47]. CK2-dependent phosphorylation of the co-chaperone CDC37, has been shown to be necessary for HSP90-CDC37 heterocomplex stabilization with client protein kinases (reviewed in [48]). As treatment with D11 resulted in down-regulation of HSP90 client proteins, we analyzed the association between HSP90 and CDC37 by *in situ* proximity ligation assay. As shown in Fig. 3c, cell exposure to D11 resulted in significant decrease in signal intensity with respect to control cells suggesting that the observed decrease in expression of components of the PI3K signaling pathway may be due to destabilization of HSP90-CDC37 heterocomplex.

#### NF-κB pathway

**Fig. 4:**
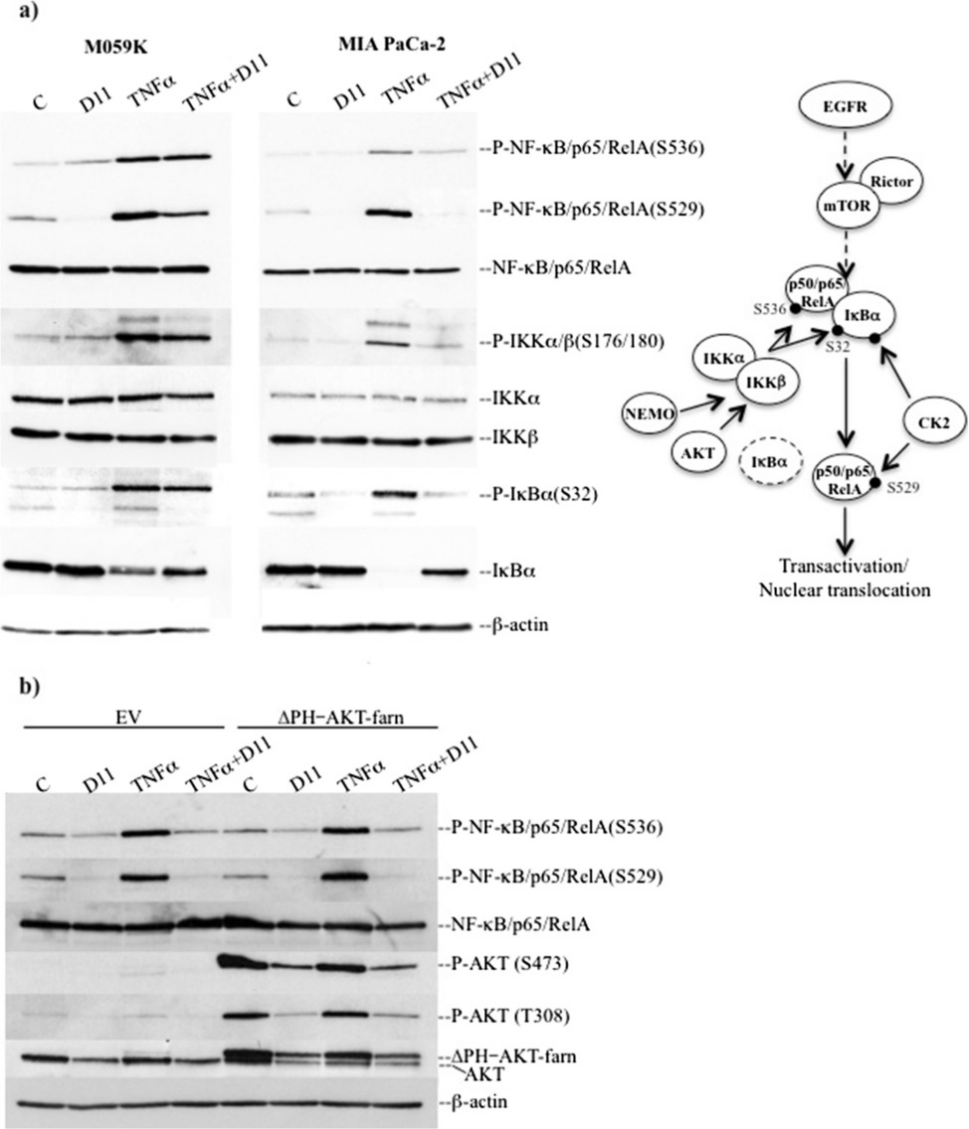
Cell incubation with D11 impairs activation of the NF-κB signaling pathway induced by TNFα. **a** Whole cell lysates from cells treated with 50 μM (M059K) and 70 μM D11 (MIA PaCa-2), respectively, for 5 h and stimulated with 20 ng/ml TNFα for 10 min prior harvesting, were analyzed by Western blot. The expression and phosphorylation levels of members of the NF-κB signaling pathway are shown. **b** MIA PaCa-2 cells transiently transfected with either empty vector (EV) or a construct containing farnesylated AKT devoid of the PH domain (ΔPH-AKT-farn) were treated as described in (**a**). Expression and activation of NF-κB as well as AKT were verified by Western blot analysis of whole cell lysates. β-actin detection was used as loading control. The figure shows a schematic model of the canonical NF-κB signaling pathway

**Fig. 5:**
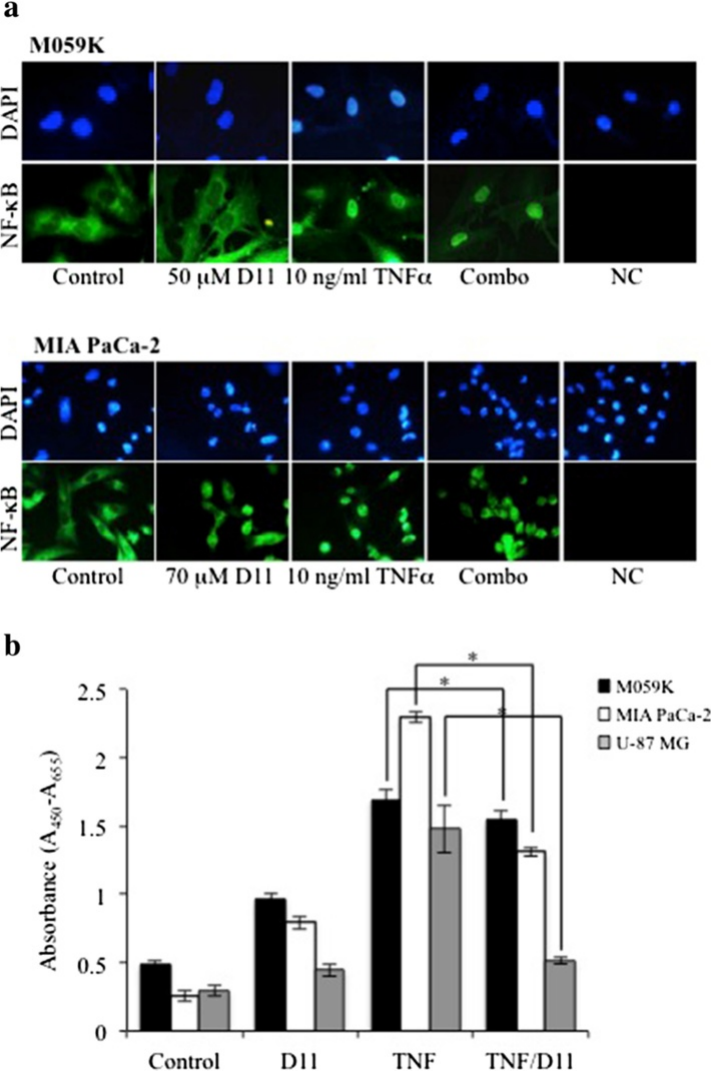
D11-mediated effects on the transcriptional activity of NF-κB. **a** Cells were treated as described in Fig. 4 and Additional file 4: Figure S4. Subcellular localization of NF-κB was detected by immunofluorescence staining of cells with rabbit monoclonal anti-NF-κB antibody (green fluorescence, Alexa Fluor 488). Nuclei were visualized by DAPI staining. NC, negative control refers to cells stained with biotinylated secondary antibody and Alexa Fluor 488 streptavidin, only. Original magnification: 400x. **b** NF-κB transcriptional activity was determined as described in Materials and methods. The indicated cell lines were treated as described in Fig. 4. Asterisks denote statistical significant differences in TNFα-induced NF-κB activity in the absence or presence of D11 (*N* = 6, *, *P* <0.05). Experiments were repeated at least three times obtaining similar results

NF-κB comprises a family of proteins existing as homo- or heterodimers whose aberrant expression and/or activity have been associated with various types of solid tumors (reviewed in [49]). Within the canonical activation pathway, the NF-κB signaling is controlled by the tumor necrosis factor receptor leading to activation of IκB kinase (IKK) complex, which in turn phosphorylates IκBα. It follows IκBα degradation and activation of NF-κB (reviewed in [50]). D11 treatment in combination with TNFα led to decreased phosphorylation of NF-κB/p65 at S536 and S529 amino acid residues as well as the upstream IKKα/β at S176/180. Interestingly, D11 treatment almost completely reversed IκBα degradation induced by TNFα suggesting impaired TNFα-mediated activation of the NF-κB pathway (Fig. 4a and Additional file 4: Figure S4). NF-κB activation downstream of EGFR-dependent signaling is regulated by mechanisms involving directly AKT and the TORC2 (Rictor-mTOR) complex, respectively [51–53]. AKT functions through IKK to promote the phosphorylation and transactivation potential of NF-κB [54]. Moreover, it has been shown that the AKT-dependent TORC1 (Raptor-mTOR)/IKK interaction stimulates IKK activity which results in the phosphorylation of IκBα and activation of NF-κB [55]. We analyzed whether overexpression of constitutively active AKT reversed D11-mediated inhibition of NF-κB in cells treated with TNFα. As shown in Fig. 4b, the expression of constitutively active AKT (farn-AKT) was unable to reverse NF-κB inhibition as the levels of IKKβ-mediated phosphorylation of NF-κB at S536 as well as the CK2-mediated phosphorylation of S529 were essentially the same in cells transfected with empty vector or a vector expressing farn-AKT. As D11 is not a direct inhibitor of AKT, mTOR, IKKα/β [17, 33, 34, 56], results reported above suggest that D11 treatment inhibits the NF-κB-dependent signaling by targeting molecule(s) other than the aforementioned proteins. Next, we analyzed whether D11 affects NF-κB migration and transcriptional activity induced by TNFα. Exposure of cells to TNFα for 10 min promptly resulted in NF-κB nuclear localization in both cell lines (Fig. 5a). However, additional treatment with D11 inhibited significantly TNFα-induced translocation of NF-κB in MIA PaCa-2 cells and only marginally in the case of M059K cells. To further support these results, we analyzed effects of D11 on the transcriptional activity of NF-κB induced by TNFα. D11 treatment significantly inhibited the transcriptional activity of NF-κB in MIA PaCa-2 and U-87 MG cells (Fig. 5b). However, consistent with results reported in Fig. 5a, a modest but significant inhibition was observed in the case of M059K cells.

### Cell incubation with D11 impairs cell migration and correlates with down-regulation of the Na^+^-K^+^-2Cl^−^ co-transporter (NKCC1)

**Fig. 6:**
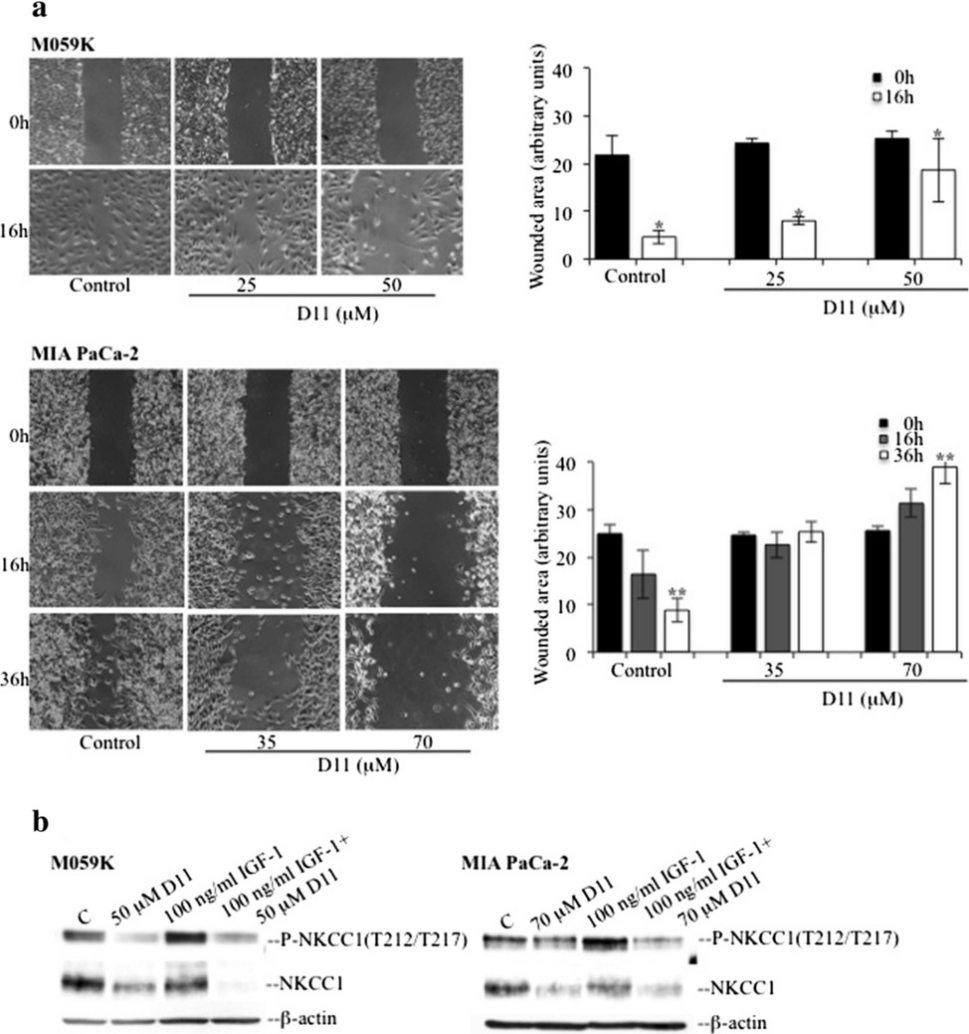
D11 treatment results in impaired cell migration and decreased expression of NKCC1. **a** M059K and MIA PaCa-2 cells were treated with vehicle or D11 as indicated in the figure and subjected to wound-healing assay. D11 was added to the cells 4 h before the wounds were made. Unwounded areas were measured as described in the Materials and methods (*N* = 6, *, *P* <0.05, **, *P* <0.0001). Original magnification: 50x. **b** Cells were treated with D11 essentially as described in Fig. 4. Stimulation with IGF-1 was performed for 15 min. β-actin detection was carried out as loading control. Experiments were repeated three times obtaining similar results

Stimulation of EGFR controls migration through activation of the PI3K/AKT signaling cascade (reviewed in [36, 37, 57–59]). Interestingly, AKT has been shown to control cell motility through phosphorylation and activation of WNK kinases (With No K-lysine), which regulate the ion co-transporter NKCC1 a modulator of migration in glioma cells [17, 57, 60–63]. As treatment of cells with D11 resulted in decreased EGFR expression levels, we assessed whether this correlated with decreased cell migration. A wound-healing assay was carried out measuring cell-unwounded areas after varying incubation times with D11 according to the speed of cell migration. Results reported in Fig. 6a show that the presence of D11 significantly impaired the ability of both cell types to migrate. In the case of M059K cells, treatment with 50 μM D11 led to about 27 % decrease in the wounded area 16 h post-wounding. In control experiments, the wounded area decreased about 79 %. Control MIA PaCa-2 cells decreased the wounded area 65 % at 36 h post-wounding while treatment with 70 μM D11 for 36 h led to approx. 52 % increase in the wounded area suggesting impaired migration and, additionally, cell death induction. Next, NKCC1 phosphorylation levels were determined in cells left untreated or exposed to D11, IGF-1 or a combination of both, respectively. Results in Fig. 6b show that D11 treatment led to decreased phosphorylation of NKCC1 at T212/217, the major activating site in NKCC1 [17, 58, 64], while EGFR activation by the pro-migratory IGF-1 [60, 62, 65, 66] did not reverse effects induced by the presence of D11. However, analysis of NKCC1 expression indicated that D11 treatment leads to significant down-regulation of NKCC1 protein expression levels.

## Discussion

*EGFR* gene amplification and mutation occur with high frequency in glioma (40–63 %) and pancreatic cancer (30–50 %). NF-κB, typically the p50-RelA/p65 heterodimer, is frequently activated in the aforementioned types of cancer resulting in enhanced cell proliferation and suppression of apoptosis. Two common mechanisms appear to contribute to NF-κB activation: i) deletion of the *NFKBIA* gene which encodes IκBα and ii) EGFR amplification. Given the importance of the EGFR and NF-κB signaling in the control of cell proliferation, survival and motility, substantial evidence suggests that these pathways may be useful targets for treatment.

In this study, we have tested the ability of a small molecule compound, D11, previously identified through a large screening of a small molecule compound library, to induce cell death in glioblastoma and pancreatic cancer cells and explored effects induced on the aforementioned signaling pathways. We show that D11 treatment results in caspase-dependent activation of cell death, which correlates with increased activity of ROCK kinase. This is in line with previous data showing that caspase 3-cleaved ROCK1, phosphorylates myosin light chains that is a necessary event for the formation of dynamic membrane protrusions consistently observed in apoptosis [17, 33, 34, 56, 67]. Analysis of the actin cytoskeleton revealed actin filaments remodeling following D11 treatment characterized by the formation of a cortical ring of actin filaments at the cell periphery accompanied by the disappearance of central stress fibers (Fig. 2c). Surma et al., demonstrated that ROCK1 and ROCK2 play different roles in the regulation of stress fiber dynamics, ROCK1 being responsible for the disruption of central stress fibers in doxorubicin-treated cells [36, 37, 57]. Similarly, data reported here suggest that actin cytoskeleton rearrangement induced by D11 is mediated by ROCK1 activation.

Treatment with D11 led to inhibition of the EGFR/PI3K signaling cascade. Protein expression analysis indicated that one of the reported effects was a significant down-regulation of HSP90-client proteins such as EGFR, PTEN, mTOR, Raptor and Tuberin/TSC2. D11 has been shown to be an effective inhibitor of protein kinase CK2 [17]. CK2 targets CDC37 for phosphorylation contributing to the stabilization of the HSP90-CDC37 heterocomplex and recruitment of client proteins. In situ proximity ligation-based analysis revealed attenuation of HSP90-CDC37 interaction in cells treated with D11 suggesting proteasome-mediated destabilization and degradation of client proteins.

We evaluated effects mediated by D11 on the expression and activity levels of AMPK. The striking result that emerged from this analysis was the finding that short exposure to the compound leads to inhibition of AMPK although a partial recovery occurs after 24 h treatment (Fig. 3b). Defective AMPK activation has been shown in some cancer cell types suggesting AMPK stimulation as a novel therapeutic approach for cancer [58]. Paradoxically, we detected a basal activity level of AMPK in both brain and pancreatic cancer cells suggesting that active AMPK is essential for sustaining proliferation of these cell lines. In support of our findings, recent studies have shown that human glioblastoma cells, astrocytes and prostate cancer cells carry activated AMPK that is required to maintain cancer cell proliferation [60, 62]. Altogether, these data indicate that the use of activators or inhibitors of AMPK as a therapeutic approach in tumor cells has to be carefully evaluated according to the biology of cancer cells.

The canonical NF-κB pathway was affected by D11 treatment. Data reported here show that D11 does reduce the TNFα-stimulated transcriptional activity of NF-κB and the extent of inhibition depends on the cell type. As D11 is not a direct inhibitor of IKKα/β [17], analysis of components of the aforementioned pathway suggests that inhibition of NF-κB results from D11-mediated targeting of EGFR, CK2 and, possibly, NEMO (IKKγ). Moreover, it cannot be excluded that the HSP90-CDC37 heterocomplex might contribute to the regulation of NF-κB as these proteins are part of the IKK complex and required for TNFα-induced IKK activation (reviewed in [65]).

Compelling evidence has underlined the importance of the EGFR and NF-κB pathways in the regulation of tumor cell migration and invasion, the latter by regulating the expression of matrix metalloproteinases [67]. We found that D11 treatment significantly impairs migration of glioblastoma and pancreatic cancer cells that correlated with decreased expression levels of NKCC1 co-transporter. This is consistent with results reported in previous studies underlining the role of NKCC1 in the modulation of glioblastoma cell volume and motility [57]. NKCC1 has been shown to be an HSP90 interacting protein suggesting that decreased expression of the co-transporter might result from disruption of the HSP90-CDC37 heterocomplex and subsequent destabilization of NKCC1 client protein [15, 68].

Our group recently reported the identification of another protein kinase CK2 inhibitor, E9, showing cytotoxic effects on human hepatoma (HepG-2) and pancreatic carcinoma (Panc-1) cell lines with corresponding IC_50_ values of 19 and 30 μM, respectively, [69]. In the future, as one cannot exclude variability in cytotoxicity with different cultured cell lines, it will be interesting to compare effects induced by E9 and D11, respectively, with the cell lines employed in this study.

## Conclusions

Overall, we report evidence that D11 treatment induces cell death and inhibits signaling pathways deregulated in brain and pancreatic cancer cells, notably, the EGFR and NF-κB signaling cascades. Exposure to D11 leads to: i) down-regulation of EGFR expression levels, which may represent a clear advantage for the treatment of certain types of cancers carrying aberrant expression of this growth factor receptor and ii) inhibition of cell motility particularly important in the case of highly invasive cancer cells [16, 70]. Data reported here underline the therapeutic potential of this compound warranting further evaluation with respect to multi-drug resistant cancer cells.

## Additional files

Additional file 1: Figure S1. Viability analysis of U-87 MG cells. Cells were treated with variable concentrations of D11 for the indicated times. The proportion of viable cells is expressed in arbitrary units as a difference in absorbance measured at 450 nm and 690 nm (reference) wavelengths, respectively. Control cells were incubated with vehicle (0.1 % DMSO). Asterisks denote statistical significant differences between control, 25 μM and 50 μM D11-treated cells for each time point, respectively, (*N* = 6, *, *P* <0.0001). (JPEG 30 kb) Additional file 2: Figure S2. FACS analysis of M059K and Mia PaCA-2 cells. Histogram representation of the Flow cytometry analysis of cells treated as described in Fig. 1b. Quantification is shown in Fig. 1b. (JPEG 61 kb) Additional file 3: Figure S3. Effect of D11 on NCS-induced cytotoxicity. (a) M059K cells were treated with D11 and NCS as indicated in the figure. Control experiment refers to cells treated with 0.1 % DMSO. Viability was determined as described in Materials and methods. Values represent mean +/− standard deviation from six samples for each treatment condition. (b) M059K whole cell lysates were subjected to Western blot analysis using an antibody against full-length and cleaved PARP. Equal loading was verified by β-actin detection. (JPEG 42 kb) Additional file 4: Figure S4. Western blot analysis of NF-κB protein expression and phosphorylation levels. Whole cell extracts from U-87 MG cells treated with vehicle or 50 μM D11 for 5 h and stimulated with 10 ng/ml TNFα for 10 min prior to harvesting, were analyzed by immunoblotting employing antibodies against NF-κB protein or the phosphorylated form at S536 and S529, respectively. β-actin detection was used as loading control. (JPEG 26 kb)
