# Supplementary figures and images for: The kinase inhibitor D11 induces caspase-mediated cell death in cancer cells resistant to chemotherapeutic treatment
Source: J Exp Clin Cancer Res. 2015 Oct 20;34:125. doi: 10.1186/s13046-015-0234-6 (PMC4612421; doi:10.1186/s13046-015-0234-6)

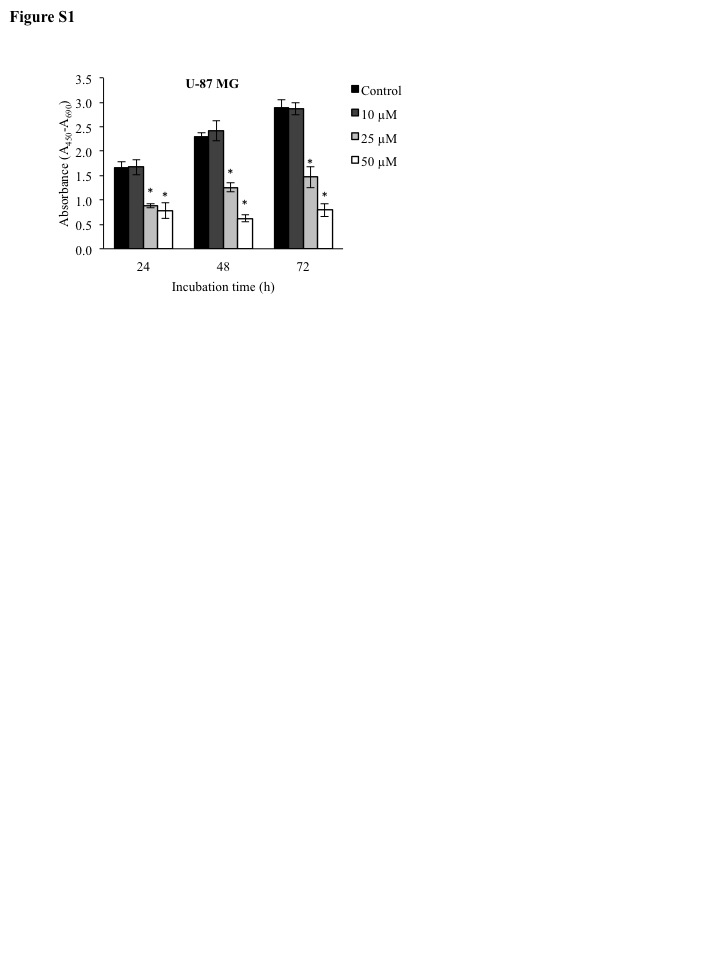

Supplement: Additional file 1: Figure S1. — Viability analysis of U-87 MG cells. Cells were treated with variable concentrations of D11 for the indicated times. The proportion of viable cells is expressed in arbitrary units as a difference in absorbance measured at 450 nm and 690 nm (reference) wavelengths, respectively. Control cells were incubated with vehicle (0.1 % DMSO). Asterisks denote statistical significant differences between control, 25 μM and 50 μM D11-treated cells for each time point, respectively, (N = 6, *, P <0.0001). (JPEG 30 kb) [file 13046_2015_234_MOESM1_ESM.jpg]

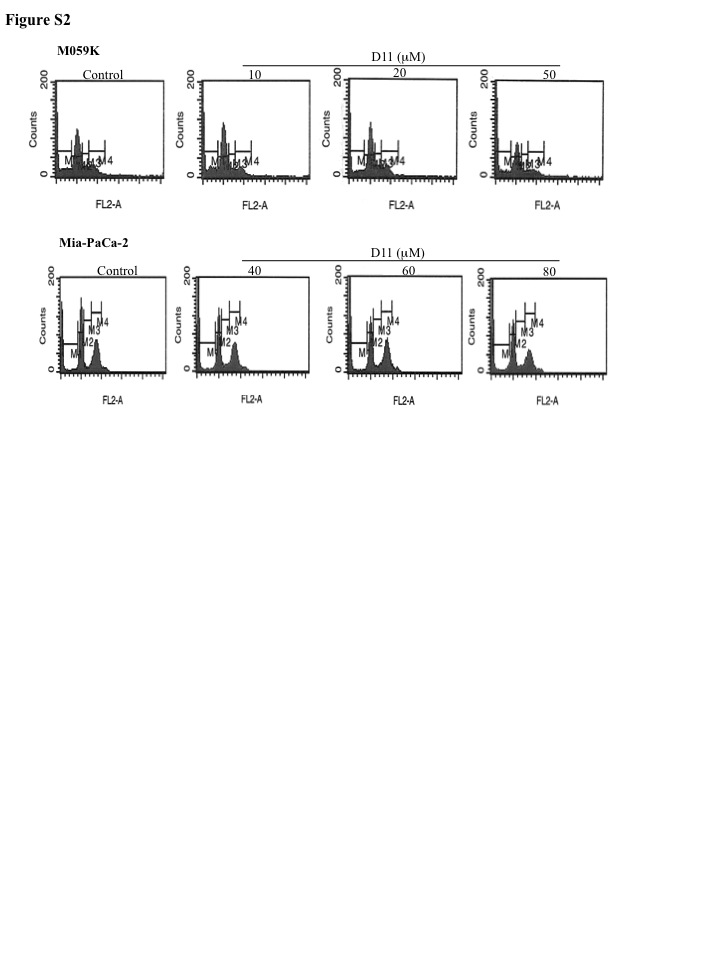

Supplement: Additional file 2: Figure S2. — FACS analysis of M059K and Mia PaCA-2 cells. Histogram representation of the Flow cytometry analysis of cells treated as described in Fig. 1b. Quantification is shown in Fig. 1b. (JPEG 61 kb) [file 13046_2015_234_MOESM2_ESM.jpg]

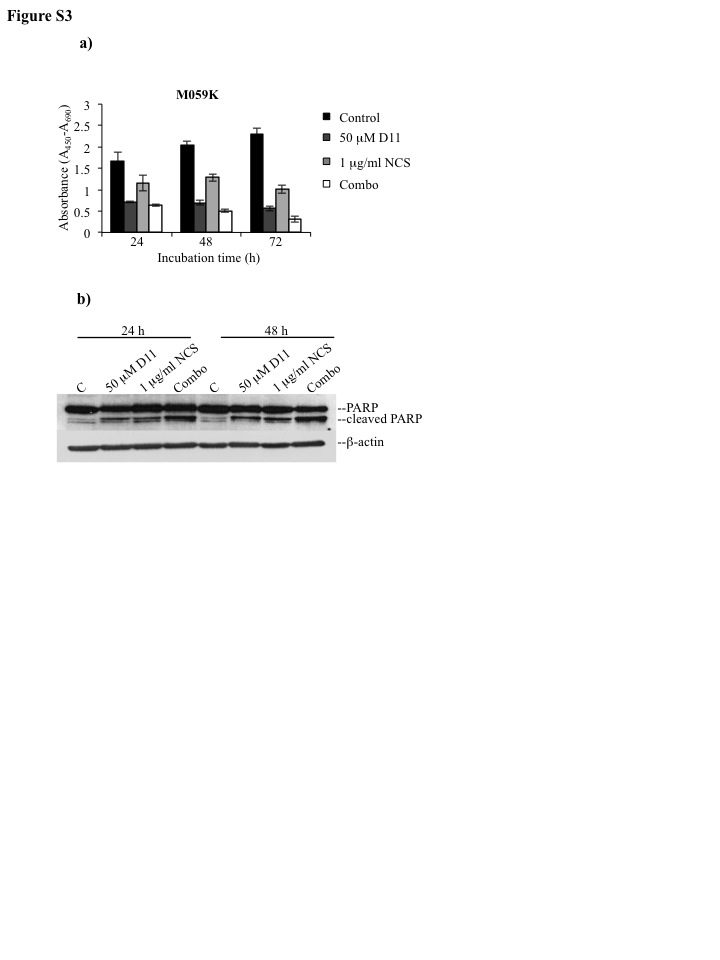

Supplement: Additional file 3: Figure S3. — Effect of D11 on NCS-induced cytotoxicity. (a) M059K cells were treated with D11 and NCS as indicated in the figure. Control experiment refers to cells treated with 0.1 % DMSO. Viability was determined as described in Materials and methods. Values represent mean +/− standard deviation from six samples for each treatment condition. (b) M059K whole cell lysates were subjected to Western blot analysis using an antibody against full-length and cleaved PARP. Equal loading was verified by β-actin detection. (JPEG 42 kb) [file 13046_2015_234_MOESM3_ESM.jpg]

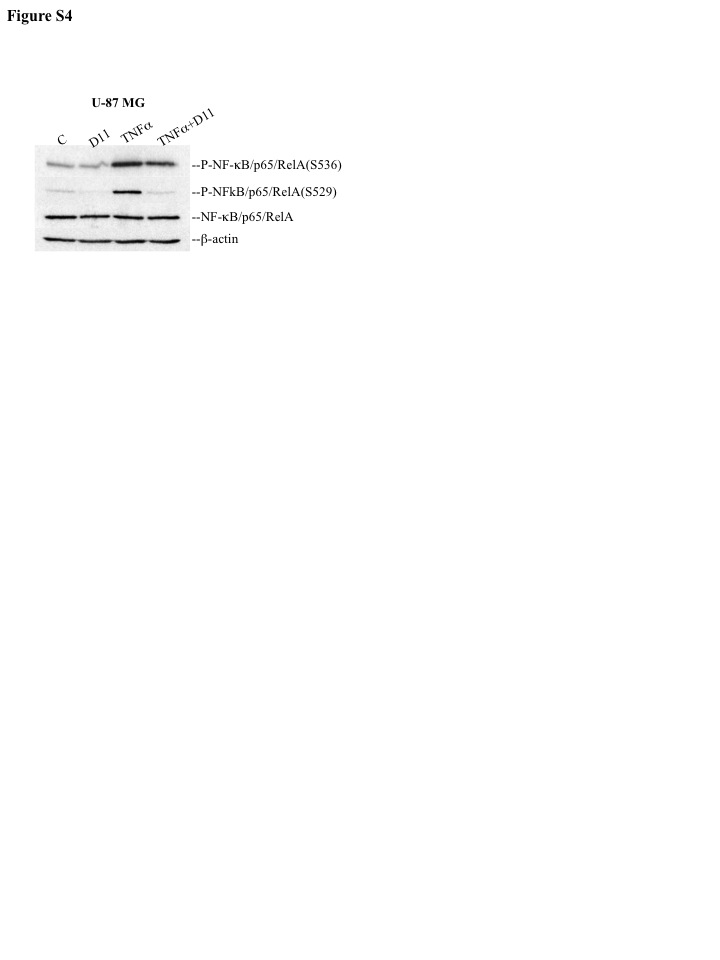

Supplement: Additional file 4: Figure S4. — Western blot analysis of NF-κB protein expression and phosphorylation levels. Whole cell extracts from U-87 MG cells treated with vehicle or 50 μM D11 for 5 h and stimulated with 10 ng/ml TNFα for 10 min prior to harvesting, were analyzed by immunoblotting employing antibodies against NF-κB protein or the phosphorylated form at S536 and S529, respectively. β-actin detection was used as loading control. (JPEG 26 kb) [file 13046_2015_234_MOESM4_ESM.jpg]
